# Tiling Nussinov’s RNA folding loop nest with a space-time approach

**DOI:** 10.1186/s12859-019-2785-6

**Published:** 2019-04-24

**Authors:** Marek Palkowski, Wlodzimierz Bielecki

**Affiliations:** West Pomeranian University of Technology, Faculty of Computer Science, Zolnierska 49, Szczecin, 71-210 Poland

**Keywords:** RNA folding, Loop tiling, Space-time tiling, Nussinov’s algorithm, Parallel computing

## Abstract

**Background:**

An RNA primary structure, or sequence, is a single strand considered as a chain of nucleotides from the alphabet AUGC (adenine, uracil, guanine, cytosine). The strand can be folded onto itself, i.e., one segment of an RNA sequence might be paired with another segment of the same RNA sequence into a two-dimensional structure composed by a list of complementary base pairs, which are close together with the minimum energy. That list is called RNA’s secondary structure and is predicted by an RNA folding algorithm. RNA secondary structure prediction is a computing-intensive task that lies at the core of search applications in bioinformatics.

**Results:**

We suggest a space-time tiling approach and apply it to generate parallel cache effective tiled code for RNA folding using Nussinov’s algorithm.

**Conclusions:**

Parallel tiled code generated with a suggested space-time loop tiling approach outperforms known related codes generated automatically by means of optimizing compilers and codes produced manually. The presented approach enables us to tile all the three loops of Nussinov’s recurrence that is not possible with commonly known tiling techniques. Generated parallel tiled code is scalable regarding to the number of parallel threads – increasing the number of threads reduces code execution time. Defining speed up as the ratio of the time taken to run the original serial program on one thread to the time taken to run the tiled program on P threads, we achieve super-linear speed up (a value of speed up is greater than the number of threads used) for parallel tiled code against the original serial code up to 32 threads and super-linear speed up scalability (increasing speed up with increasing the thread number) up to 8 threads. For one thread used, speed up is about 4.2 achieved on an Intel Xeon machine used for carrying out experiments.

## Background

Ribonucleic acid (RNA) molecule is one of the most important molecules in the biological systems. RNA is typically produced as a single stranded molecule, which then folds intramolecularly to form a number of short base-paired stems. This base-paired structure is called the secondary structure of the RNA. The dynamic programming approach to RNA secondary structure prediction relies on the fact that structures can be recursively decomposed into smaller components. In each of the decomposition steps, only a single loop (or stacking of two consecutive base pairs) needs to be evaluated.

Nussinov proposed a dynamic programming algorithm for RNA folding in 1978 [1], which maximizes the number of non-crossing matchings between complimentary bases of an RNA sequence of length *N*.

Let *X*=*x*_1_,*x*_2_,…,*x*_*N*_ be an RNA sequence, where *x*_*i*_∈{*G*(*guanine*),*A*(*adenine*),*U*(*uracil*),*C*(*cytosine*)} is a nucleotide. Nussinov’s dynamic programming recurrence for *N*×*N* matrix *S* is given below.

$$S(i,j) = \max\limits_{1 \leq\ i < j \leq N} \left\{\begin{array}{l} S(i+1, j-1) + \sigma(i,j)\\ \max\limits_{i \leq k < j}(S(i, k) + S(k + 1, j)), \end{array}\right. $$

Here, *S*(*i,j*) defines the maximum number of base-pair matches of *x*_*i*_,…,*x*_*j*_ over the region 1≤ *i*<*j*≤*N* and *σ*(*i,j*) is a function, which returns 1 if (*x*_*i*_,*x*_*j*_) is an *AU*, *GC*, or *GU* pair and 0 otherwise.

Listing 1 represents the triply nested affine loops with two statements accessing the two-dimensional array *S* implementing Nussinov’s algorithm.


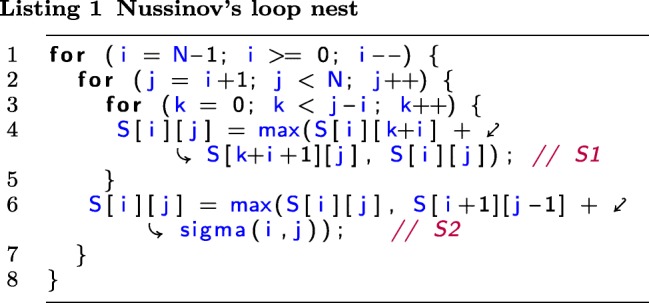


Fast Nussinov implementations for shared memory architectures must ensure both aspects of code parallelism and cache optimization. Cache optimization is not found or limited to the memory layout optimization to improve spatial locality in popular parallel implementations of RNA folding, for example, GTFold [2], UNAfold [3] or RNAfold [4], which, however, implement energy minimization.

There are several manual or empirical approaches in literature improving data locality of serial or multi-threaded RNA folding code, e.g. [5–10], dedicated to various hardware platforms including GPUs and FPGAs.

Li et al. [5] suggested a cache efficient version of Nussinov’s recurrence by using the lower triangle of matrix *S* to store the transpose of the computed values in the upper triangle of *S*. As new *S*_*i,j*_s are computed, they are stored in both *S*_*i,j*_ and *S*_*j,i*_ for *j*≤*i*. The sum *S*_*i,k*_+*S*_*k*+1,*j*_ is computed as *S*_*i,k*_+*S*_*j,k*+1_. Hence, Li’s modifications accelerate rapidly code execution because reading values in a row is more cache efficient than reading values in a column [5].

Zhao and Sahni developed three cache-efficient algorithms without increasing the memory requirement, *ByRow*, *ByRowSegment*, and *ByBox* for Nussinov’s RNA folding [6]. They showed that presented techniques based on a simple LRU cache model give better run time and energy performance than Li’s approach. Unfortunately, no parallel code is presented by the authors.

The effectiveness of automatic tiling and parallelization of loop nests depends a lot of their dependence patterns. The dependences of a loop nest can be classified into two categories: uniform and non-uniform. The dependences are uniform only when the distances between dependent loop nest statement instances in the iteration space are uniform, i.e., these distances are expressed by constants; otherwise they are non-uniform. A set of distance vectors represents distances between dependent loop nest statement instances calculated as the difference between the iteration vectors representing the destinations and sources of dependences.

For uniform dependences, the corresponding dependence graph is regular while for non-uniform dependences it is irregular. Automatic tiling and parallelization of loop nests with non-uniform dependences by means of affine transformations is much difficult than those exposing uniform dependences. The reason is that for such dependences, in general, constraints formed to extract affine transformations (to be next applied to tile and parallelize loops) are parametric and non-linear, this considerably increases the computational complexity of extracting affine transformations. Even when affine transformations can be found, they do not guarantee efficient loop tiling and parallelization: only some loops from all ones in the loop nest can be tiled and/or parallelized.

The Nussinov kernel involves mathematical operations over affine control loops whose iteration space can be represented by the polyhedral model [11]. However, the Nussinov RNA folding acceleration is still a challenging task for modern compilers because that code is within nonserial polyadic dynamic programming (NPDP), which is a particular family of dynamic programming with non-uniform data dependences, and it, as mentioned above, is more difficult to be optimized [7].

Optimizing compilers usually apply loop tiling to generate cache-efficient code on multicore architectures that maximizes data reuse in deep memory hierarchies and reduces synchronization cost [12]. Loop tiling transformations allow for improving data locality and generate coarse-grained parallel code that leads to improving code performance [13]. The most popular techniques of tiling are based on the affine-transformation framework (ATF), which is implemented in several tools [12].

Pluto [14] is the most popular state-of-the-art source-to-source polyhedral code generator that transforms C programs to parallel coarse-grained code with enhanced data locality. Pluto uses a scheduling algorithm, which tries to find affine transformations allowing the most efficient tiling. The main purpose is to minimize the amount of inter-tiles communications and ensure parallelism among tiles. The Pluto schedule is optimal, it reduces the number of dependences crossing tile boundaries. Unfortunately, Pluto fails to generate a band of loops where all multiple consecutive loops may be interchanged while respecting the legality in the case of rectangular tiling for NPDP kernels. Pluto serializes the innermost loop of Nussinov’s RNA folding, which is a key of cache locality optimization [15]. As a consequence, Pluto fails to generate 3-D tiles that prevents achieving maximal code locality and performance.

Mullapudi and Bondhugula introduced a dynamic tiling technique for Zuker’s RNA secondary structure prediction [11]. Their technique overcomes some limitations of the affine transformation framework. Generated tiles are of the 3-D dimension and they can be scheduled only at run-time, i.e., that technique does not allow for generation of any static code.

Wonnacott et al. suggested 3-D tiling of “mostly-tileable” loop nests representing Nussinov’s RNA secondary structure prediction [16]. This approach extracts non-problematic statement instances in the loop nest iteration space, i.e., those that can be safely tiled by means of well-known techniques. The reminding statement instances should be run serially to preserve all the dependences available in the loop nest. Unfortunately, the approach allows for generation of only serial code.

The tiling technique presented in paper [15] transforms (corrects) original rectangular tiles into target ones, which are valid under lexicographic order. Tile correction is performed by means of the transitive closure of loop dependence graphs. Loop skewing is used to parallelize code. We achieved higher speed-up of generated tiled code in comparison with that produced with state-of the-art source-to-source optimizing compilers. However, the correction technique generates irregular tiles, some of them can be too large, this does not allow us to achieve maximal code locality and performance [17].

In this paper, we present a novel approach of space-time tiling for accelerating Nussinov’s RNA folding, which allows for generation of tiled code with the following features:

- the dimension of generated tiles is 3-D,
- generated code can be easily parallelized by means of the skewing technique,
- target parallel code is scalable regarding to the number of threads and the length of an RNA sequence,
- generated parallel code is regular and compact, it outperforms known automatically and manually generated related codes.

**Fig. 1:**
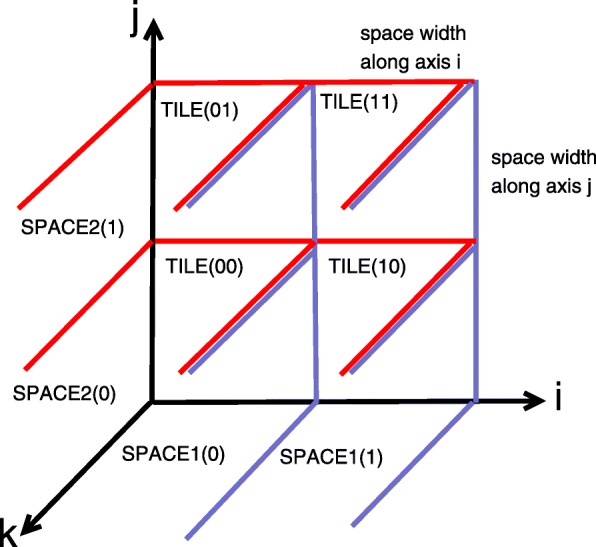
Spaces and tiles

The concept of space-time tiling is the following. Scrutinizing the Nussinov loop nest, we discover that dependences along both axis *i* and *j* spread in only the forward direction, i.e., the two corresponding elements of all dependence distance vectors are non-negative (taking into account that the value of index *i* is decremented). Using this fact, we split the iteration spaces of the loop nest statements into two groups of sub-spaces of a fixed width, which intersect axes *i* and *j* and are in parallel with planes (*j,k*) and (*i,k*), respectively. Figure 1 presents such sub-spaces for statement S1. Blue lines depict planes situated in parallel with plane (*j, k*) while red ones present planes located in parallel with plane (*i, k*). Each sub-space of the first and second groups is represented with identifiers *id*_1_ and *id*_2_, respectively; integers in brackets are the identifiers of sub-spaces; name *SPACEi*(*j*) states for the sub-space belonging to group *i,i*=1,2 and its identifier is *j*=0,1.

Then we form tiles as the intersection of sets representing the sub-spaces mentioned above, see Fig. 1. Each such a tile is represented with an identifier (*id*_1_,*id*_2_), where *id*_1_ and *id*_2_ are the identifiers of the corresponding sub-spaces, in Fig. 1, they are shown in brackets. Such tiles are valid under lexicographical order because inter-tile dependences are spread in only the forward directions regarding to both *id*_1_ and *id*_2_, i.e, the both corresponding elements of all dependence distance vectors are non-negative.

**Fig. 2:**
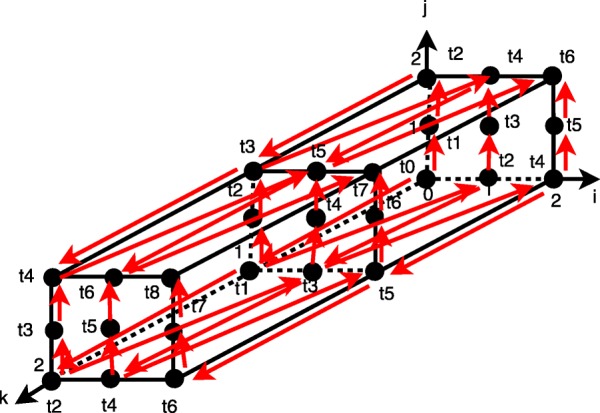
Time slices

The size of generated tiles is limited along axes *i* and *j* with the width of sub-spaces, but it is not limited along axis *k* that reduces code locality – see Fig. 1. To split each tile generated as presented above into sub-tiles along axis *k*, we can find any valid schedule of statement instances allowing for forming time partitions, which are to be enumerated serially, while statement instances of each time partition can be run in parallel. Let us consider Fig. 2. It represents the iteration space of a single tile provided that the sub-space width is equal to 3. Suppose that dependences within that tile are characterized with the following set of distance vectors.

{(0,0,1),(0,1,0),(1,0,−1)}.

Distance vectors allow us to generate all dependences in the iteration space shown in Fig. 2. To find the destination of a dependence with a given dependence source within the iteration space, we add a distance vector to this source and if the resulting iteration is within the iteration space, we conclude that there is the dependence in that iteration space.

In Fig. 2, we show with red arrows some dependences corresponding to the distance vectors above. Valid time partitions, *ti*, can be represented with the following sets, each including iterations, which can be run in parallel.

*t*0:={ (0,0,0) },

*t*1:={ (0,0,1),(0,1,0)},

*t*2:={ (0,0,2),(0,1,1),(0,2,0),(1,0,0) },

*t*3:={ (0,1,2),(0,2,1),(1,0,1),(1,1,0) },

*t*4:={ (0,2,2),(1,0,2),(1,1,1),(1,2,0),(2,0,0) },

*t*5:={ (1,1,2),(1,2,1),(2,0,1),(2,1,0) },

*t*6:={ (1,2,2),(2,0,2),(2,1,1),(2,2,0) },

*t*7:={ (2,1,2),(2,2,1) },

*t*8:={ (2,2,2) }.

Each time partition comprises independent iterations, which can be executed in parallel while time partitions should be enumerated in lexicographical order. In Fig. 2, labels *ti*,*i*=0,1,2,…,8 mark a time partition comprising the corresponding iteration.

Next we combine time partitions into time slices each including a fixed number of time partitions. Provided that the number of time partitions in a slice is equal to 3, we get the following time slices, *TIMEi, i*=1,2,3.

*TIME1*:=*t*0 ∪ *t*1 ∪ *t*2={ (0,1,2),(0,2,1),(1,0,1),(0,0,1),(1,1,0),(0,1,0),(0,0,0) },

*TIME2*:=*t*3 ∪ *t*4 ∪ *t*5={ (0,2,2),(1,1,2),(0,1,2),(1,0,2),(1,2,1),(0,2,1),(1,1,1),(2,0,1),(1,0,1),(1,2,0),(2,1,0),(1,1,0),(2,0,0) },

*TIME3*:=*t*6 ∪ *t*7 ∪ *t*8={ (2,2,2),(1,2,2),(2,1,2),(2,0,2),(2,2,1),(2,1,1),(2,2,0) }.

Enumerating time slices is valid under lexicographic order because before executing the next slice all data necessary are already calculated in the previous slices. So, within a single tile, we form sub-tiles represented with time slices whose execution in serial order increases code locality because instead of execution of one larger tile, multiple smaller sub-tiles will be executed.

To generate target tiles, we apply the intersection operation to sets representing sub-spaces and time slices. In the following section, we prove that for the Nussinov loop nest, resulting tiles are valid under lexicographical order and demonstrate how parallel target code can be generated.

## Methods

In compiler research, polytopes and related mathematical objects have been successfully applied to represent and manipulate an important class of compute- and data-intensive programs in an approach that has become known as the *polyhedral model*. It formalizes analyzing, parallelizing, and transforming program fragments consisting of (sequences of) arbitrarily nested loops (like dynamic programming loops), where the loop bounds, statements conditions and array accesses are affine combinations of symbolic constants and loop iterators.

The polyhedral method treats each iteration of a loop statement within the loop nest as an integral point inside mathematical objects called polyhedra that contains all iterations of the statement. A convex polyhedron can be formally defined as the set of solutions to a system of linear inequalities of the form *Mx*≤*b*.

The polyhedral model of a loop nest includes i) a set representing an iteration space for each statement, ii) access relations (read and write) for each array available in the loop nest body, iii) relations describing a global schedule for each statement – a discrete time when a statement instance is executed according to the original iteration execution order.

A mathematical representation of a set is the following.

*S*:=*PARAMS*→{*NAME*(*I*) | *constraints*}, where *S* is the set name, “ *PARAMS*→” means that the *constraints* include parameters *PARAMS*, each parameter is an arbitrary integer whose value defines an upper loop bound, *NAME*(*I*) is the named tuple with name *NAME* and vector *I* whose elements are loop indices or expressions including loop indices; *constraints* are comprised of affine equations and inequalities including vector *I* and parameters *PARAMS* combined through the conjunction (∧), disjunction (∨), projection (∃), and negation (¬) operators. For example, *S*:=*N*→{*S*1(*i,j*)| 1<=*i*<=*N* ∧ 1<=*i*<=*N*} denotes a parametric set (regarding to parameter *N*) with name *S*, named tuple *S*1(*i,j*), and constraints 1<=*i*<=*N* ∧ 1<=*i*<=*N*.

Relations are defined in a similar way as sets, except that a single tuple is replaced with a pair of tuples separated by the arrow sign “ →”, i.e, the mathematical representation of a relation is the following.

*R*:=*PARAMS*→{*NAME1*(*I*)→*NAME2*(*J*)|*constraints*}, where *R* is the relation name, *NAME1*(*I*) and *NAME2*(*J*) are the named tuples with names *NAME1* and *NAME2* including vectors *I* and *J*, respectively. For example, *R*:=*N*→{*S*1(*i*1,*j*1)→*S*2(*i*1,*j*2)| 1<=*i*1,*i*2<=*N* ∧ 1<=*i*1,*j*2<=*N*} denotes the parametric relation with name *R*, tuples *S*1(*i*1,*j*2),*S*2(*i*2,*j*2) and the constraints 1<=*i*<=*N*1<=*i*1,*i*2<=*N* ∧ 1<=*i*1,*j*2<=*N*. A relation maps elements represented with the first tuple to elements of the second one.

To extract the polyhedral model for a C program (all its three components), the PET (Polyhedral Extraction Tool) tool can be applied [18]. To extract dependences available in a loop nest, we use the polyhedral model returned with PET and apply the iscc calculator [19] to implement calculations on polyhedral sets and relations in a way presented in paper [20]. Iscc is an interactive interface to the barvinok counting library [21] and PET.

To optimize code, we use the following operations on relations and sets: intersection (∩), union (∪), difference (-), domain (dom *R*), range (ran *R*), relation application (*S* ^′^=*R*(*S*): *e* ^′^∈*S* ^′^ iff exists *e* s.t. *e* →*e* ^′^∈*R*,*e* ∈*S*) [21].

Given the loop nest, the iteration *i* is lexicographically less than iteration *j*, denoted as *i*≺*j*, if the following conditions are true *i*_1_<*j*_1_∨∃*k*≥1:*i*_*k*_<*j*_*k*_∧*i*_*t*_=*j*_*t*_,*for* *t* <*k*.

The iteration domain of the Nussinov loop nest in Listing 1) is represented with the following parametric set comprising all the statement instances executed for statements S1 and S2.

*Iteration* *Domain*:=*N*→{*S*1(*i,j,k*) | 0≤*i*≤*N*−−1∧ *i*+1≤*j*≤*N*−−1∧ 0≤*k*≤*j*−*i*−1;*S*2(*i,j*) | 0≤*i*≤*N*−−1∧ *i*+1≤*j*≤*N*−−1},

where *S*1(*i,j,k*),*S*2(*i,j*) are the tuples defining the iteration domain of the first and second statements of the Nussinov loop nest, respectively; the constraints of the tuples specify the value range of indices *i, j, k* in the loop nest.

The relation representing dependences available in the examined loop nest is the following.

*R*:=*N*→{ S1(*i, j, k*)→S2(*i*^′^,*i*+*j*−*i*^′^)∣0≤*k*<−*i*+*j*∧*i*^′^≥−1+*i*∧*i*^′^≥0∧−*N*+*i*+*j*<*i*^′^≤*i* }∪*N*→{ S1(*i*,*j*,*k*)→S1(*i*,*j*,*k*^′^)∣*i*≥0∧*j*<*N*∧*k*≥0∧*k*<*k*^′^<−*i*+*j* }∪*N*→{ S1(*i*,*j*,*k*)→S1(*i*,*j*^′^,−*i*+*j*)∣*i*≥0∧0≤*k*<−*i*+*j*∧*j*<*j*^′^<*N* }∪*N*→{ S1(*i*,*j*,*k*)→S1(*i*^′^,*j*,−1+*i*−*i*^′^)∣*j*<*N*∧0≤*k*<−*i*+*j*∧0≤*i*^′^<*i* }∪*N*→{ S2(*i*,*j*)→S1(*i*,*j*^′^,−*i*+*j*)∣*i*≥0∧*j*>*i*∧*j*<*j*^′^<*N* }∪*N*→{ S2(*i*,*j*)→S1(*i*^′^,*j*,−1+*i*−*i*^′^)∣*i*<*j*<*N*∧0≤*i*^′^<*i* }∪*N*→{ S2(*i*,*j*)→S2(−1+*i*,1+*j*)∣*i*>0∧*i*<*j*≤−2+*N* },

where “ *N*→” means that *N* is a parameter, i.e., an arbitrary constant whose value defines loop upper bounds in the relation constraints. Relation *R* is represented with a union (∪) of simpler relations included in curly braces. Each simple relation includes two named tuples, for example, S1(…) →S2(…) and constraints. Elements of tuples are loop iterators or affine expressions whose components are loop iterators. The left tuple defines dependence sources while the right one states for the corresponding dependence destinations. Name S1 of the left tuple means that dependence sources are originated with statement S1 while S2 marks that dependence destinations are descended with statement S2. Each constraint includes affine inequalities whose elements are loop iterators and parameter *N*, for example, 0≤*k*<−*i*+*j*, multiple inequalities are combined by means of the conjunction operator (∧).

Using relation *R*, we can discover the maximal number of rectangular sub-space types to be formed, this number is defined with the maximal number of the outer loops for which all elements of all distance vectors (formed as the differences between the range and domain of relation *R*) are non-negative. Such subspaces can be enumerated in lexicographical order because there is no cycle among them (the two corresponding elements of all distance vectors are non-negative).

Because statements S1 and S2 have different iteration spaces, we should form a global iteration space common for both statements S1 and S2. With this purpose, we apply the global schedule of the statements of the examined loop nest. It is a part of the loop nest polyhedral model and represented with a relation, which maps an iteration vector of a statement to a corresponding multidimensional timestamp, i.e., a discrete time when the statement instance has to be executed in the common iteration space. PET returns the following global schedule for the examined loop nest.

*SCHED*_*GLOB*:=*N*→{ S2(*i, j*)→(*i*,*j*,1,0) }∪*N*→{ S1(*i*,*j*,*k*)→(*i*,*j*,0,*k*) }.

Next, we form relation, *R*_*GLOB*, by means of replacing each named tuple of relation *R* with the tuple resulting due to applying relation *SCHED*_*GLOB* to named tuples *S*_1_ and *S*_2_. That relation describes dependences in the common iteration space and it is as follows.

*R*_*GLOB*:=*N*→{ (*i*,*j*,0,*k*)→(*i*,*j*,*k*^′^,1,0)∣*i*≥0∧*j*<*N*∧*k*≥0∧*k*<*k*^′^<−*i*+*j* }∪*N*→{ (*i*,*j*,0,*k*)→(*i*^′^,*i*+*j*−*i*^′^,1,0)∣0≤*k*<−*i*+*j*∧*i*^′^≥−1+*i*∧*i*^′^≥0∧−*N*+*i*+*j*<*i*^′^≤*i* }∪*N*→{ (*i*,*j*,0,*k*)→(*i*,*j*^′^,0,−*i*+*j*)∣*i*≥0∧0≤*k*<−*i*+*j*∧*j*<*j*^′^<*N* }∪*N*→{ (*i*,*j*,0,*k*)→(*i*^′^,*j*,0,−1+*i*−*i*^′^)∣*j*<*N*∧0≤*k*<−*i*+*j*∧0≤*i*^′^<*i* }∪*N*→{ (*i*,*j*,1,0)→(*i*,*j*^′^,0,−*i*+*j*)∣*i*≥0∧*j*>*i*∧*j*<*j*^′^<*N* }∪*N*→{ (*i*,*j*,1,0)→(*i*^′^,*j*,0,−1+*i*−*i*^′^)∣*i*<*j*<*N*∧0≤*i*^′^<*i* }∪*N*→{ (*i*,*j*,1,0)→(−1+*i*,1+*j*,1,0)∣*i*>0∧*i*<*j*≤−2+*N* }.

Then we apply the *deltas* operator of the iscc calculator to relation *R*_*GLOB* and get the following distance vectors in the global (common) iteration space.

*N*→{ (*i*,−*i*,1,*k*)∣−1≤*i*≤0∧2−*N*−2*i*≤*k*≤0 }∪*N*→{ (*i*,0,0,*k*)∣*i*<0∧−*N*−2*i*<*k*<−*i* }∪*N*→{ (0,*j*,0,*k*)∣*j*>0∧0<*k*<*N*−*j* }∪*N*→{ (0,*j*,−1,*i*_3_)∣*j*>0∧0<*i*_3_<*N*−*j* }∪*N*→{ (*i*,0,−1,−1−*i*)∣2−*N*≤*i*<0 }∪*N*→{ (−1,1,0,0)∣*N*≥4 }.

Taking into account that the value of iterator *i* is decremented, we conclude that only the third element of each distance vector is negative. This allows us to state that sub-spaces located in parallel with plane (*j,k*) and intersecting axis *i* as well as sub-spaces located in parallel with plane (*i,k*) and intersecting axis *j*, can be enumerated in lexicographical order because the both elements of all the corresponding dependence distance vectors are non-negative.

We split the iteration spaces of statements S1 and S2 into sub-spaces of width 16 (any other constant width can be chosen). For statement S1, sets *SPACE*_11_ and *SPACE*_12_ below represent sub-spaces intersecting axes *i* and *j*, respectively.

*SPACE*_11_:=(*ii*,*N*)→{ S1(*i*,*j*,*k*)∣*ii*≥0∧*N*>0∧*i*≥0∧−16−16*ii*+*N*≤*i*<−16*ii*+*N*∧*i*≤−2+*N*∧*i*<*j*<*N*∧0≤*k*<−*i*+*j* },

*SPACE*_12_:=(*jj*,*N*)→{ S1(*i*,*j*,*k*)∣*jj*≥0∧*N*>0∧0≤*i*≤−2+*N*∧*j*>16*jj*+*i*∧*i*<*j*<*N*∧*j*≤16+16*jj*+*i*∧0≤*k*<−*i*+*j* }.

For statement S2, sets *SPACE*_21_ and *SPACE*_22_ below represent sub-spaces intersecting axes *i* and *j*, respectively.

*SPACE*_21_:=(*ii*,*N*)→{ S2(*i*,*j*)∣*ii*≥0∧*N*>0∧*i*≥0∧−16−16*ii*+*N*≤*i*<−16*ii*+*N*∧*i*≤−2+*N*∧*i*<*j*<*N* },

*SPACE*_22_:=(*jj*,*N*)→{ S2(*i*,*j*)∣*jj*≥0∧*N*>0∧0≤*i*≤−2+*N*∧*j*>16*jj*+*i*∧*i*<*j*<*N*∧*j*≤16+16*jj*+*i* }.

Variables *ii* and *jj* are the parametric identifiers of sub-spaces. The intersection of the union of sets *SPACE*_11_, *SPACE*_12_ and the union of sets *SPACE*_21_, *SPACE*_22_ results in tiles whose size is limited along axes *i* and *j* with the width of sub-spaces (16), but the size of those tiles is not limited along axis *k*. Fig. 1 presents such sub-spaces and tiles for statement S1, integers in brackets are the identifiers of sub-spaces and tiles. Blue lines depict planes situated in parallel with plane (*j, k*) while red ones present planes located in parallel with plane (*i, k*).

For large *N*, the entire data associated with each such a tile cannot be held at cache, this leads to decreasing code locality. To improve code locality, we form time slices each including a constant number of time partitions. Each time partition holds statement instances that can be run in parallel for a given schedule. For a time slice, the number of statement instances within axes *k* is limited with a constant number of time partitions within that slice. Let us suppose that a time slice is represented with a parametric set, *TIME*, while a set representing the results of the intersection of above mentioned sets is named as *SPACE*. Then the intersection of sets *TIME* and *SPACE* results in a parametric set describing tiles whose size is limited along all axes: *i, j*, and *k*. Choosing the proper width of sub-spaces and the proper number of time partitions within a time slice, we may obtain tiles for which the entire data associated with each of them can be held at cache, this can improve significantly code locality.

To form time partitions, we apply the loop skewing transformation [22]. It is a convenient method to implement the wavefront method of executing a loop nest in parallel, which creates a “wave” that passes through the iteration space. Skewing changes the iteration vectors for each iteration by adding the outer loop index value to the inner one, for example, for a loop nest of depth 2 with iterators *i* and *j*, iteration (*i,j*) becomes relabeled as (*i,i*+*j*). If all distance vectors of a new loop nest with iterators *i* and *i*+*j* comprise only non-negative elements, interchanging those iterators, i.e., forming new iterators *i*+*j* and *i* allows for generation of a new loop nest, where the outermost loop *i*+*j* is serial while *i* is parallel. In general, for a loop nest of depth *d*, a new loop nest with iterators (*i*_1_+*i*_2_+…+*i*_*d*_,*i*_1_,*i*_2_,…,*i*_*d*−1_) is valid if all distance vectors comprise only non-negative elements; the first loop is serial while the reminding ones are parallel [22].

For the Nussinov loop nest, we first form the following schedule.

*SCHED*:=*N*→{ S1(*i, j, k*)→(−*i*+*j*,*k*)∣*N*>0∧0≤*i*≤−2+*N*∧*i*<*j*<*N*∧0≤*k*<−*i*+*j* }∪*N*→{ S2(*i*,*j*)→(−*i*+*j*,*j*)∣*N*>0∧0≤*i*≤−2+*N*∧*i*<*j*<*N* }.

That schedule maps each instance of statements S1 and S2 to two-dimensional time (−*i*+*j*,*k*) and (−*i*+*j*,*j*), respectively. To check whether that schedule is valid, we apply the way suggested in paper [23], which envisages checking whether the following inequality *δ*((*SCHED*^−1^). *R*. *SCHED*)≥ 0 is true, where *R* is the relation describing all the dependences available in the examined loop nest, it is presented above; “.” is the iscc join (composition) operator of two relations; *δ* is the *deltas* iscc operator that maps a relation to the differences between image and domain elements.

Using such a checking, we conclude that relation *SCHED* above is valid. By means of relation *SCHED*, we form set *TIME* representing time slices each including 16 time partitions (any other constant value can be chosen). With this purpose, we first calculate the inverse relation, *SCHED*^−1^, of relation *SCHED*.

*SCHED*^−1^:=*N*→{ (*i*0,*i*1)→S10(*i*,*i*0+*i*,*i*1)∣*N*>0∧*i*_0_>0∧0≤*i*_1_<*i*0∧0≤*i*<*N*−*i*0∧*i*≤−2+*N* }∪*N*→{ (*i*_0_,*i*_1_)→S12(−*i*0+*i*1,*i*1)∣*N*>0∧*i*_0_>0∧*i*0≤*i*_1_<*N*∧*i*_1_≤−2+*N*+*i*0 }.

In the relation above, variables *i*0,*i*1 represent two-dimensional time. To form set *TIME*, we i) make *i*0 to be the parameter of set *TILE*, ii) make the right tuple and the constraints of relation *SCHED*^−1^ to be the tuple and constraints of set *TIME*, iii) introduce parameter *tt* of set *TIME* and add the constraints of the form *i*1≥16*tt*∧0≤*i*1≤15+16*tt* to the constraints of set *TIME*, those constraints mean that the width of a time slice is 16 (any other constant value can be chosen) and time partitions within a time slice are dependent on parameter *tt*. This results in the following set.

*TIME*:=(*i*0,*tt*,*N*)→{ S1(*i*,*i*0+*i*,*i*1)∣*i*0>0∧*N*>0∧0≤*i*<−*i*0+*N*∧*i*≤−2+*N*∧*i*1≥16*tt*∧0≤*i*1≤15+16*tt*∧*i*1<*i*0 }∪(*i*0,*tt*,*N*)→{ S2(*i*,*i*0+*i*)∣*i*0>0∧*N*>0∧*i*≥0∧−*i*0+16*tt*≤*i*<−*i*0+*N*∧*i*≤15−*i*0+16*tt*∧*i*≤−2+*N* }.

We calculate set *TILE*, which represents target tiles, as follows

*TILE*:=*TIME*. (*SPACE*_11_∪*SPACE*_12_). (*SPACE*_21_∪*SPACE*_22_)=(*N, jj, ii, i0, tt*)→{ S2(*i*,*i*0+*i*)∣*jj*≥0∧*N*>0∧*ii*≥0∧*i*0>16*jj*∧0<*i*0≤16+16*jj*∧*i*≥−16+*N*−16*ii*∧*i*≥0∧−*i*0+16*tt*≤*i*≤15−*i*0+16*tt*∧*i*<*N*−16*ii*∧*i*<*N*−*i*0∧*i*≤−2+*N* }∪(*jj*,*N*,*ii*,*i*0,*tt*)→{ S1(*i*,*i*0+*i*,*k*)∣*jj*≥0∧*N*>0∧*ii*≥0∧*i*0>16*jj*∧0<*i*0≤16+16*jj*∧*i*≥−16+*N*−16*ii*∧0≤*i*<*N*−16*ii*∧*i*<*N*−*i*0∧*i*≤−2+*N*∧*k*≥16*tt*∧0≤*k*≤15+16*tt*∧*k*<*i*0 }.

To find out what is the number of statement instances within a tile represented with the set above, we applied the iscc *card* operator to set *TILE*, which calculates the number of elements within a set. The analysis of the result returned with that operator allows us to conclude that the size of each tile is not parameterized, i.e., the number of elements within each tile does not depend on the parameter *N* defining the upper bounds in the Nussinov loop nest as it takes place when the tile correction technique [15] is applied to generated target tiles.

Let us re-write set *TILE* in the following form.

*TILE*:=(*N, jj, ii, i0, tt*)→{ S1(*i*,*i*0+*i*,*k*)∣ *constraints*_1_; S2(*i*,*i*0+*i*)∣ *constraints*_2_ }.

To generate parallel code on the tile level, we apply the skewing transformation (*ii*+*jj*) to form the following schedule allowing for parallel code generation.

*SCHED*_*PAR*:=*N*→{ S1(*i*,*i*0+*i*,*k*)→(*ii*+*jj,jj,i0,tt, i, i0*+*i, k*)∣ *constraints*_1_;S2(*i*,*i*0+*i*)→(*ii+jj,jj,i0,tt, i, i0*+*i*)∣ *constraints*_2_},

where *constraints*_1_ and *constraints*_2_ are the constraints of set *TILE* above.

That schedule maps each instance of statements S1 and S2 to a time partition whose all tiles can be executed in parallel. To check the validity of that schedule we again apply the technique presented in paper [23] and explained above to relation *SCHED*_*PAR*, and confirm that the schedule is valid.

Applying the iscc *codegen* operator to relation *SCHED*_*PAR*, we generate pseudocode and postprocess it to the parallel tiled code presented in Listing 2. In that code, the outermost loop *c*0 enumerates serially time partitions including tiles, loop *c*1 scans tiles to be executed in parallel for each time partition, loops *c*3,*c*4, and *c*6 enumerate serially statement instances within each tile, *pragma omp parallel for* makes loop *c*1 parallel [24].


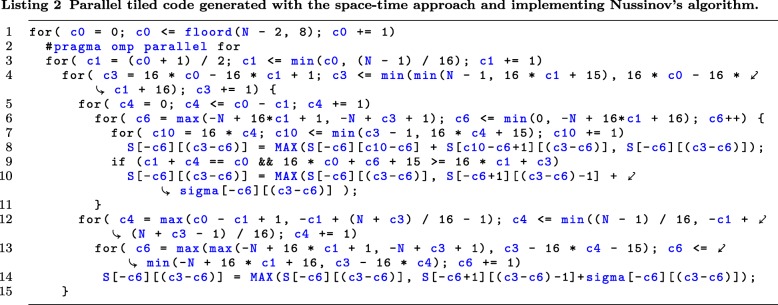


## Results

We conducted experiments on RNA secondary structure prediction using a machine with 2 × Intel Xeon processors E5-2695 v2, 2.4 GHz, 12 cores/24 threads, 256KB L2 Cache and 30MB L3 Cache, and 128 GB RAM. All programs were compiled with using of the Intel C++ Compiler (*icc* 17.0.1) with the -O3 flag of optimization. The code parallelism is presented with the OpenMP programming interface [24].

At the address traco.sourceforge.net/nuss.tar.gz , all source codes used for carrying out experiments can be found as well as a program allowing us to run each parallel program for a prepared sequence in the FASTA format and obtain a target Nussinov table.

To carry out experiments, we used randomly generated RNA strands of length from 2500 to 15000. Papers [5, 6, 15] show that cache efficient code performance does not change based on strings themselves, but it depends on the size of a string.

We compared the performance of code generated with the presented approach with that of i) Pluto parallel tiled code (based on affine transformations), ii) tiled code based on the correction technique [15, 17], and iii) the Li manual cache efficient implementation [5] of Nussinov’s RNA folding. The tile size 16 ×16×1 for Pluto code [14] was chosen empirically (Pluto does not tile the most inner loop) as the best among many sizes examined. For the code generated with the presented approach, the tile size 16 ×16×16 was chosen from many different tile sizes, examined by us, as one exposing the highest code performance. We used this tile size also for tiled code generated with the tile correction technique based on the transitive closure of dependence graphs. We experimented with tiled code based on correction also for the best tile size demonstrated in paper [17], 1 ×128×16.

**Fig. 3:**
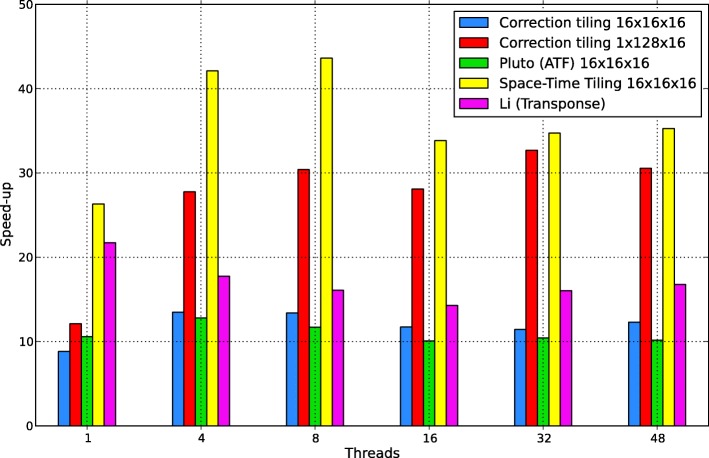
Speed up of the examined codes implementing Nussinov’s RNA folding depending on the number of threads

The results in Table 1, including the execution time of the examined programs and Fig. 3 graphically presenting the corresponding code speed up against the original code, demonstrate that only the code generated with the suggested approach is scalable up to 48 threads, i.e., increasing the number of threads reduces the time of code execution. The results in Table 1 show that space-time tiled code implementing Nussinov’s algorithm with the tile size [16 ×16×16] outperforms all examined implementations for a larger number of threads (equal or greater than 16) for *N*=5000.

**Table 1:** Execution times of the examined codes depending on the thread number for *N*=5000

*N*=5000	Original	Tile correction	Tile correction	Pluto (ATF)	Space-Time Tiling	Li (Transpose)
Threads		16 ×16×16	1 ×128×16	16 ×16×1	16 ×16×16	
1	243.4322	132.3891	77.4809	130.748	57.7063	66.56
4		57.5215	19.5745	64.9885	21.634	21.24
8		38.4351	11.8201	32.2481	12.563	14.05
16		18.455	7.8852	18.662	7.1172	12.39
32		17.3659	7.9378	23.1456	6.8839	12.44
48		16.0771	8.7662	19.0443	5.7779	13.72

**Fig. 4:**
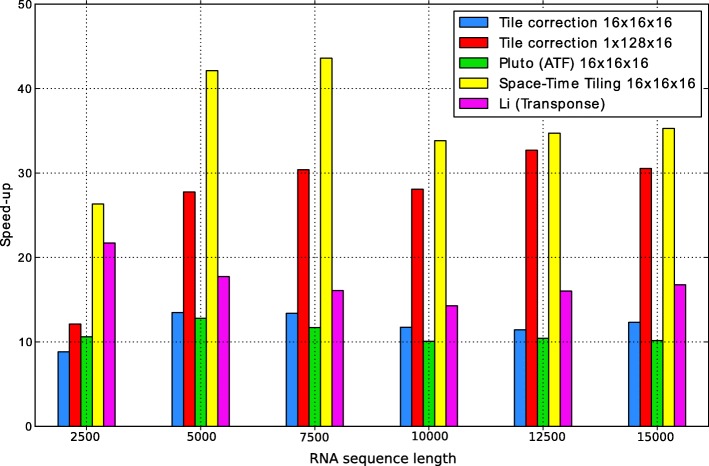
Speed up of the examined codes depending on the RNA sequence length for 48 threads used

Table 2 and Fig. 4 present execution time and speed up for various RNA sequence lengths, respectively. We can see that the presented approach allows for obtaining cache efficient tiled code, which outperforms the other examined implementations for each length.

**Table 2:** Execution times of the examined codes depending on the RNA sequence length for 48 threads used

48 Threads	Original	Tile correction	Tile correction	Pluto (ATF)	Space-Time Tiling	Li (Transpose)
Seq. length		16 ×16×16	1 ×128×16	16 ×16×1	16 ×16×16	
2500	21.06	2.39	1.74	1.99	0.80	0.97
5000	243.43	18.08	8.77	19.04	5.78	13.72
7500	812.10	60.72	26.72	69.49	18.62	50.50
10000	1709.43	145.66	60.86	169.76	50.52	119.82
12500	3450.01	302.01	105.50	331.21	99.35	215.20
15000	5863.83	476.65	192.01	577.76	166.29	349.76

## Discussion

In paper [17], we showed that tile correction allows for generation code of the best performance when the outermost loop is serial. We revealed that the best tile size (for tile correction) is not optimal for the code generated with the suggested approach in that case, tiling all loops is a better solution.

Space-time tiled code achieves super-linear speed up (greater than the number of threads used) of serial and parallel tiled code against the original serial code up to 32 threads. However super linear speed up scalability (increasing speed-up with increasing the thread number) is observed only from one to eight threads. Code regularity and lack of parametric large tiles allow us to use the entire power of the multi-processor machine with the maximal number of threads while the speed up of Li’s code and that based on tile correction is significantly limited in this case.

The transposition of the Nussinov array [5] is effective only for short RNA strands with the length equal to 2500. For longer sequences (greater than 7500), only the performance of code based on applying transitive closure with the best tile size is comparable, but it is still worse than the performance of code generated with the approach presented in this paper.

Summing up, we may conclude that the parallel tiled code generated by means of the presented space-time approach is the fastest implementation among all examined ones including the manual generated Li code. Code regularity and fixed tiles are dominant factors in achieving high code performance and scalability in comparison to our previous implementation based on tile correction. We achieved the best code performance for each RNA sequence length using all cores/threads on the studied Intel Xeon machine.

## Conclusion

In this paper, we introduced a space-time tiling approach for the loop nest implementing Nussinov’s folding. It allows us to generate parallel tiled code, which outperforms known related codes generated automatically by means of affine transformations, tile correction based on the transitive closure of dependence graphs, and manually generated Li’s transposition code. The presented approach enables us to tile all three loops of Nussinov’s recurrence that is not possible with commonly known optimizing compilers based on affine transformations. The approach generates 3-D tiles for the Nussinov loop nest.

The results of an experimental study allow us to conclude that the generated code based on the space-time approach i) outperforms known related codes, ii) is scalable regarding to the number of parallel threads (execution time decreases with increasing the thread number up to 48 threads); iii) allows for achieving super-linear speed up (greater than the number of threads used) of serial and parallel tiled code against the original serial code up to 32 threads and super-linear speed up scalability (increasing speed up with increasing the thread number) up to 8 threads, for one thread, speed up is about 4.2.

The presented code optimization can be applied to other dynamic programming kernels, for example, DNA sequence alignment or energy minimization for RNA folding. In the future, we plan to apply space-time tiling to Zuker’s algorithm allowing for energy minimization. It is much complex than the algorithm examined in this paper – there are four nested loops and multiple complicated statements within the corresponding loop nest. However that code is still within the polyhedral model, so space-time tiling can be applied to that code to tile all loops.

## Abbreviations

ATF: Affine transformation framework
FPGA: field-programmable gate array
NPDP: Nonserial polyadic dynamic programming
